# Determination of Tropifexor in Beagle Dog Plasma by UPLC-MS/MS and Its Application in Pharmacokinetics

**DOI:** 10.1155/2022/2823214

**Published:** 2022-09-17

**Authors:** Yan-Ding Su, Xin-Yi Wei, Xiao-Hang Su, Ghulam Woshur, Xiao-Nan Geng, Xiang-Jun Qiu

**Affiliations:** School of Basic Medical Sciences, Henan University of Science and Technology, Luoyang 471023, China

## Abstract

The primary objective of this study was to develop and validate an efficient and accurate ultra-performance liquid chromatography-tandem mass spectrometry (UPLC-MS/MS) approach as a means to detect tropifexor plasma concentrations in beagle dogs and to study its pharmacokinetic profile in beagle dogs. The chromatographic separation of tropifexor and oprozomib (internal standard, ISTD) on the column, with the addition of acetonitrile for rapid precipitation and protein extraction, was achieved with 0.1% formic acid aqueous solution-acetonitrile for the mobile phase. A Xevo TQ-S triple quadrupole tandem mass spectrometer, under the selective reaction monitoring (SRM) mode, for the determination of the concentrations in the positive ion mode. The mass transfer pairs of tropifexor and oprozomib (ISTD) were m/z 604.08 ⟶ 228.03 and m/z 533.18 ⟶ 199.01, respectively. The profile displayed well linearity with calibration curves for tropifexor and oprozomib (ISTD) ranging from 1.0 to 200 ng/mL. In parallel, the lower limit of quantification (LLOQ) value for tropifexor could be measured with the aid of this novel technique at 1.0 ng/mL. In addition, the scope of intraday and interday for analyte accuracy was between −4.86% and 1.16%, with a precision of <7.31%. The recoveries of the analytes were >88.13% and were free of significant matrix effects. The stability met the requirements for the quantification of plasma samples under various conditions. Finally, the pharmacokinetic profile of tropifexor in beagle dog plasma following oral administration of 0.33 mg/kg tropifexor was determined by using the method facilitated in this work.

## 1. Introduction

The term nonalcoholic fatty liver disease (NAFLD) refers to a syndrome of pathology dominated by excessive fat deposition in the hepatocytes due to diseases other than alcohol consumption and other well-defined liver injuries. NAFLD has been described as including steatosis simplex and nonalcoholic steatohepatitis (NASH). Steatosis simplex is the accumulation of triglycerides. In contrast, NASH, as a prolonged disease, will likely progress to hepatic fibrosis, hepatocirrhosis, and eventually hepatocellular cancer if it continues to progress. Currently, the proportion of NASH in NAFLD is 10%–30% [1], which is 10% more than the data in the 2010 China NAFLD diagnosis and treatment guidelines [2]. The global prevalence of NAFLD is increasing, from 15% in 2005 to 25% in 2010. Similarly, among patients with NAFLD, the incidence of progression to NASH has almost doubled [3]. Therefore, the prevention and treatment of NASH have become a research focus as an important stage in the progression from steatosis simplex to hepatic fibrosis, hepatic cirrhosis, and liver cancer.

The pathogenesis of NASH is currently considered unclear, and the “multiple parallel strikes” theory suggests that NASH is the result of parallel interactions between multiple risk factors, multiple cell types, and multiple tissues and organs. Insulin resistance, lipotoxicity, oxidative stress, endoplasmic reticulum stress, systemic hypoinflammatory response, immune or cytokine or mitochondrial function alterations, and apoptosis are the pathogenic pathways involved in the development and progression of NASH [4]. For the treatment of NAFLD, basic therapies such as weight reduction, diet control, and exercise should be recommended first. Due to poor patient compliance, these approaches are not ideal in the management of NASH and hepatic fibrosis [5]. At the same time, drugs to treat and improve NASH were created, including insulin sensitizers, angiotensin receptor blockers, lipid-lowering drugs, antioxidants, hexoketo cocaine, and ursodeoxycholic acid. However, because of the obvious limitations of these drugs, their effectiveness and security still warrant ongoing investigation with clinical trials. So, there is a real demand for continuous research of current and potential drugs both clinically and preclinically to augment NASH therapy.

Hence, there is an urgent clinical imperative to discover new drugs against NASH, and this has led to targeted interventions targeting these pathogenic mechanisms becoming a hot topic of current research. Most of the current research is focused on single-target interventions. Theoretically, therapeutic targets need to be balanced against hepatic steatosis, inflammation, hepatocyte injury, and fibrosis. The combination of multiple drug interventions targeting different targets in the pathogenesis of NASH is a direction for future research.

Currently, there are six nuclear receptor farnesoid *X* receptor (FXR) agonists entering clinical trials worldwide for the NASH indication [6]. Tropifexor, one of the new high-potency agonists for FXR, shows an EC50 value of 0.2 nM. In addition, tropifexor observed a strong induction of BSEP and small heterodimer partner (SHP) genes among primary cells in a concentration-dependent manner. When the concentration was as low as 1 nM, we could note that the induction of BSEP was higher than that of the control (DMSO), while at 10 nM, a 15-fold stronger SHP induction than the control was observed. And at 1 nM, a moderate SHP induction, three times higher than the control, was observed [7]. In parallel, pharmacokinetic studies in rats revealed that tropifexor was poorly cleared with a CL value of 9 mL·min^−1^·kg^−1^ and it also possessed a high terminal *t*_1/2_ of 3.7 h. Tropifexor as an aqueous microemulsion formulation was formulated with an oral bioavailability of 20% in rats. In mice, administered by intravenous injection, tropifexor displayed both poor clearance and narrow volume of distribution with a half-life of 2.6 hours. In dogs, when administered intravenously, tropifexor showed a *t*_1/2_ of 7.4 hours and a volume of distribution of 0.46 L/kg [7]. Tropifexor targets FXR in the intestinal epithelial cells and causes a concentration-dependent increase in FGF-19 levels in single- and multiple-dosing experiments [8]. In a model of ANIT-induced liver injury, tropifexor could ameliorate hepatic transcarbamylase and fibrosis [9]. Among the Stelic animal models of NASH (STAM), in which tropifexor regressed well-established fibrosis and decreased NAFLD activity scores and hepatic glycerol triglycerides. Meanwhile, tropifexor dramatically downregulated adipose hepatitis, fibrosis, and fibrogenic gene expression in the insulin-precipitated hepatic NASH model(AMLN) [10].

To date, LC-MS/MS approaches have been used for pharmacokinetic-related studies of tropifexor [11]. However, an analytical bioanalytical method for the detection of tropifexor in biological samples by ultra-performance liquid chromatography-tandem mass spectrometry (UPLC-MS/MS) has not been available. Therefore, the work aims to verify a facile and precise UPLC-MS/MS assay which measures tropifexor in the plasma of beagle dogs as well as investigate the pharmacokinetic profile of tropifexor in Beagles.

## 2. Experimental

### 2.1. Reagents and Materials

**Figure 1:**
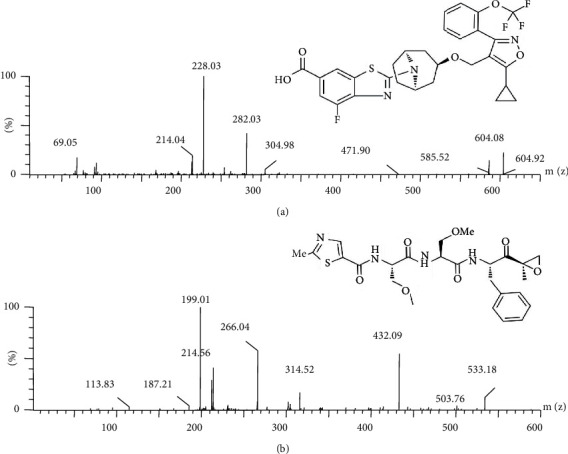
In the present work, the mass spectra of tropifexor (a) and oprozomib (b).

Tropifexor (Figure 1(b)) (purity > 98%) and Oprozomib (Figure 1(a)) (internal standard, ISTD, purity > 98%) that were sourced from Shanghai Tronsai Technology Co. HPLC grade methanol and acetonitrile which were obtained from Merck (Darmstadt, Germany). Milli-Q reagent system (Bedford Millipore, USA) followed by filtration and preparation of ultrapure water.

### 2.2. Instrumentations and Analytical Conditions

The apparatus used in the chromatographic analysis was a Waters ACQUITY ultra-performance liquid chromatography (UPLC) (Milford, Massachusetts, USA). The basic procedure was as follows. First of all, the chromatographic separation was done on a Waters ACQUITY UPLC BEH C18 (50 mm × 2.1 mm, 1.7 *μ*m) and a precolumn. Then, thorough separation of analytes was achieved by an efficient gradient process with both mobile phase acetonitrile (B) and 0.1% formic acid aqueous solution (A). The column temperature was controlled at 40°C for the entire elution period, with the autosampler (FTN) set at 10°C, and the sample chamber temperature at 4°C. The injection volume was 2.0 *μ*L and the flow rate was constant at 0.3 mL/min. The whole gradient elution process lasted for 2.0 min, and the acetonitrile concentration was 10% within 0 to 0.5 min. Then, by 1 min, the acetonitrile concentration reached 90% and was maintained up to 1.4 min, until it dropped to 10% at 1.5 min and continued up to 2.0 min.

A Waters Xevo TQ-S triple quadrupole tandem mass spectrometer (Milford, MA, USA) and an electrospray ion source (ESI) with an ESI temperature and mass spectrometry voltage of 550°C and 5500 V (positive), respectively, were used, and both were combined to perform positive ion scanning in selective reaction monitoring (SRM) mode. Finally, as summarized in Table 1, the control parameters and statistics of the MS/MS system that were acquired by Masslynx 4.1 were listed.

### 2.3. Preparation of Standard and Quality Control (QC) Samples

The weighed tropifexor and oprozomib were dissolved in methanol, fixed in a 10 mL volumetric flask, and mixed thoroughly to form a stock solution and an internal standard stock solution with a mass concentration of 1.0 mg/mL, respectively. To obtain a valid solution mixture, appropriate amounts of the reserve solution were removed and gradually diluted with methanol, and the same was done for the ISTD working solution, eventually diluting both down to 200 ng/mL. After preparing the standard working solution, 10 *μ*L of it was taken, followed by adding 90 *μ*L of blank beagle dog plasma which was configured as a standard solution of plasma. The standard concentrations for the tropifexor calibration curve were as follows: 1, 2.5, 5, 10, 25, 50, 100, and 200 ng/mL. The blank samples and different concentrations of standard working solutions were precisely aspirated to configure three concentrations of quality control (QC) samples of 2.5, 50, and 150 ng/mL as low quality control (LQC), medium quality control (MQC), and high quality control (HQC), severally. All of the above configurations were kept under 4°C for further experimental studies.

### 2.4. Sample Operation

A precise amount of 100 *μ*L of the plasma specimen was placed in a 1.5 ml plastic centrifuge tube. Then, add 20 *μ*L of ISTD working solution was added, followed by 250 *μ*L of acetonitrile to precipitate the protein. Then, the three mixtures were vortexed for 2.0 min and centrifuged at 10,000 × *g*, 4°C for 15 min. Afterward, the supernatant was pipetted into the autosampler vial with an internal insertion tube for a final injection of 2.0 *μ*L for analysis and determination.

### 2.5. Method Validation

The selectivity, standard curve, precision and accuracy, matrix effect, as well as stability for the proposed approach were verified as per “Guidelines for the Validation of Quantitative Methods for the Analysis of Biological Samples” in the fourth general rule of the Chinese Pharmacopoeia, 2020 edition.

Three different types of beagle dog samples were selected: real plasma samples obtained in pharmacokinetic studies, blank plasma adulterated with tropifexor and ISTD, and six blank beagle dog plasma samples from different batches. Separately, they were analyzed and evaluated as a screening for the selectivity of the protocol.

Using the linearity of the analytical procedure, the results were obtained in proportion to the concentration of the sample, and the tropifexor regression was calculated using a weighted (*W* = 1/*X*^2^) least squares algorithm at eight different concentrations between the scope of 1.0–200 ng/mL, based on the concentration of the test substance in the plasma as the horizontal coordinate *X* (ng/ml), and the ratio of the peak area of the test substance to that of the internal standard as the vertical coordinate *Y*. The calibration curve was established by regression with the weighted (*W* = 1/*X*^2^) least squares method. The range of LLOQ of the regression equation should be within ±20%.

Three QC samples, at three concentration levels, were used to assess accuracy (RE, %) and precision (RSD, %). Intraday precision and accuracy are assessed by six replicate samples of each concentration level analyzed in one day, as well as interday precision and accuracy over three consecutive days. The accepted values for accuracy and precision should be limited to ±15%.

The extraction recovery of this experiment was assessed by dividing the response value of the analyte recovered from the sample matrix by the response value generated by the standard at three quality control levels (2.5, 50, and 150 ng/mL). The analytes were added with concentrations at 2.5, 50, and 150 ng/mL to validate the assessment of the matrix effect (ME) by comparing the peak area from the existence of the matrix with the proportion of the corresponding one without matrix. The ME of ISTD was also assessed the same way at a working concentration of 100 ng/mL.

The stability of tropifexor was appraised at five replicate levels of 150, 50, and 2.5 ng/mL under the following stock conditions utilizing a newly prepared calibration curve. Initially, the samples were subjected to room temperature for 24 hours to ascertain their short-term stability. In addition, the long-term stability was also evaluated by storing the samples at 80°C for 60 days. Also, the freeze-thaw stability of the analytes in plasma was studied during three freeze-thaws. Eventually, the extracted samples from the sample manager (10°C) were stored for 8 hours to determine the stability of the autosampler. The final value obtained should be within ±15% of the concentration at the time of the preliminary analysis.

### 2.6. Animal Experiments and Pharmacokinetic Study

Six healthy, aged adult Beagles (2–3 years old) selected by the Experimental Animal Center of Henan University of Science and Technology (Luoyang, China), weighing between 8.0 and 10.0 kg, were selected and kept in the experimental kennel for a week of qualified feeding and ensuring their normal diet. The production license No.: SCXK(E) 2021–0020. Throughout the experimental process, the animal studies followed the Guide for Ethical Review of Laboratory Animal Welfare (GB/T35892-2018).

After fasting for more than 12 hours the day before the experiment, each beagle was given 0.33 mg/kg tropifexor prepared from 0.5% sodium carboxymethylcellulose (CMC-Na) by oral administration. Next, approximately 1.0 mL of venous blood was collected at 0, 0.25, 0.5, 1, 2, 4, 6, 8, 12, 24, and 48 hours and stored in a heparin-containing 1.5 mL polyethylene tube. The blood samples were then centrifuged at 4°C for 10 minutes at 3000 rpm. The supernatant was taken immediately after centrifugation and stored at −80°C for subsequent analysis.

The established UPLC-MS/MS method was used to detect the concentration of tropifexor in the beagle dog plasma. The drug and statistics (DAS) 2.0 software was utilized to execute a nonatrioventricular analysis to derive the primary pharmacokinetic parameters that we desired.

## 3. Results and Discussions

### 3.1. Method Development and Optimization

The standard solution (1 *μ*g/mL) was scanned for both positive and negative ion patterns under the continuous injection mode by a flow syringe pump, and the findings indicated that the [M + H]^+^ of the compound had better stability and sensitivity in the positive ion mode. The mass spectrometry parameters were automatically optimized to select the optimal target analyte spectral conditions and characteristic daughter ions.

Acetonitrile was chosen as the organic phase due to its low column pressure and high mass spectrometric response value. Then, this experiment compared the effects of water-acetonitrile, 0.1% formic acid aqueous solution-acetonitrile, and 0.1% acetic acid aqueous solution-acetonitrile on the response of the target compounds, which showed a high response and good specificity of the target analytes, when the mobile phase was 0.1% formic acid aqueous solution-acetonitrile. Meanwhile, this experiment compared Waters ACQUITY UPLC BEH C18 (50 mm × 2.1 mm, 1.7 *μ*m) with Waters ACQUITY UPLC HSS T3 column (50 mm × 2.1 mm, 1.8 *μ*m), and the results showed that the former had better separation effect than the latter and could meet the requirements of instrumental analysis. Finally, acetonitrile was chosen as the reagent for the protein precipitation method because of its lack of significant endogenous interference and high extraction rate compared to those of methanol.

### 3.2. Method Validation

#### 3.2.1. Sample Selectivity

**Figure 2:**
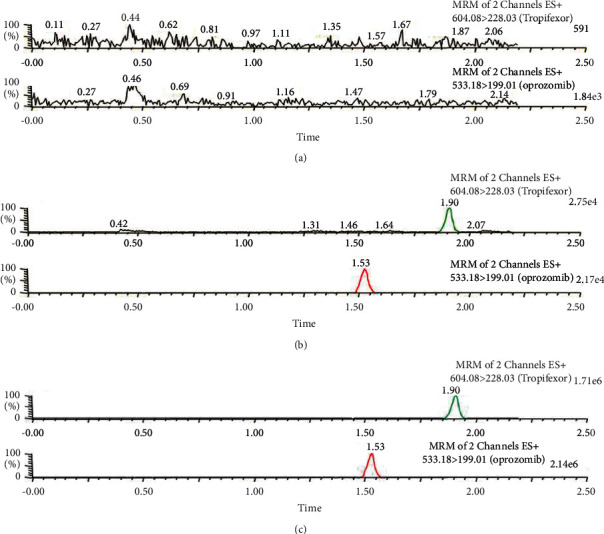
Typical chromatograms from blank plasma (a), blank plasma (b) added with standard solution, and an authentic plasma specimen (c) after the oral administration of tropifexor by beagle dogs.

We verified the selectivity of the protocol by comparing the chromatograms obtained from the blank plasma specimens of beagle dogs with those taken from the plasma samples in which the standard solution was added, as well as with those of the plasma samples from beagle dogs after the oral administration of the drug. As illustrated by Figure 2, the retention times of tropifexor and ISTD were not affected by the blank plasma samples, with retention times of 1.53 and 1.90 min for both. The outcomes point out that this investigation was reproducible with selectivity and specificity.

#### 3.2.2. Calibration Curve and LLOQ

From Table 2, it was evident that the coefficient for determinacy (*r*^2^) of the linear regression analysis remained above 0.99 throughout the validation test, while the standard curve of tropifexor was well linear in the range of 1 to 200 ng/mL. The regression equation, as verified by this study, is *Y* = 0.01921 × *X* + 0.01570 (*r*^2^ = 0.9994). Lastly, LLOQ was the minimum concentration of analyte in the sample that could be reliably quantified, and the LLOQ value for tropifexor in this study was 1.0 ng/mL, which was within 20% of the relative precision and accuracy.

#### 3.2.3. Precision and Accuracy

The accuracy and precision of tropifexor were obtained by performing multiple replicate assays at four different concentrations, as illustrated in Table 2, with in-depth analysis at LLOQ and three QC samples. The obtained accuracies and precision ranges were within ±15%. The findings suggest that this approach was reliable, as well as accurate for the measurement of tropifexor in beagle plasma.

#### 3.2.4. Matrix Effect and Recovery

In Table 3, it could be concluded that the average extraction recoveries obtained for the analytes derived from beagle plasma for QC samples at three concentration levels were 88.13%–93.13%, indicating the high reproducibility of the method. The ME of tropifexor in this work ranged from 99.88%–101.93%, indicating that no clear matrix effects were exhibited.

#### 3.2.5. Stability

After studying and analyzing the stability of tropifexor under 2.5, 50, and 150 ng/mL concentrations, the obtained outcomes were steady under short-term, long-term, freeze-thaw, and sample manager (10°C) conditions as shown in Table 4. The RSD was less than 15% for all storage conditions, which was following the requirements of the “Guidelines for the validation of quantitative methods for the analysis of biological samples” in the fourth general rule of the Chinese Pharmacopoeia, 2020 edition.

### 3.3. Pharmacokinetic Study

**Figure 3:**
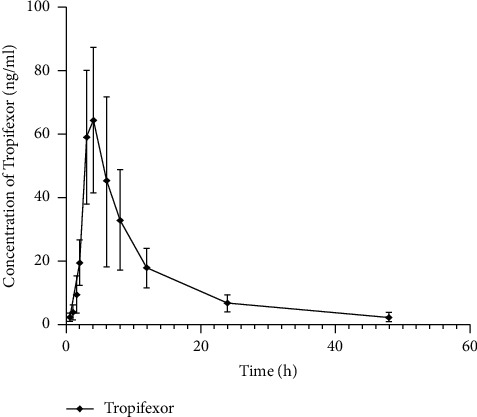
Mean plasma concentration–time curves of tropifexor in beagles (*n* = 6).

The concentration of tropifexor in plasma after oral administration of 0.33 mg/kg of tropifexor in beagle dogs was determined by a novel method, the UPLC-MS/MS technique.  Figure 3 shows the mean blood concentrations of tropifexor over time, as seen in the main pharmacokinetic parameters shown in Table 5. Tropifexor was absorbed gradually after oral administration and reached its maximum concentration (*C*_max_) within 3.33 ± 0.52 hours after dosing. Furthermore, tropifexor had a half-life (*t*_1/2_) of 9.27 ± 2.63 hours, which was comparable to the data presented in the previous study in dogs. Although, pharmacokinetic parameters of tropifexor in experimental animals were already studied, the UPLC-MS/MS technology was so far not used to study the concentration of tropifexor in Beagle dog plasma. Therefore, for the first time, we performed the UPLC-MS/MS technique to measure the pharmacokinetic parameters for tropifexor with beagle dogs.

## 4. Conclusions

In conclusion, an accurate and reliable UPLC-MS/MS method was prepared for the measurement of tropifexor in plasma as well as its pharmacokinetic levels in beagles were characterized. The optimized method was demonstrated to have low interference, good reproducibility, high accuracy, precision, and good linearity. This method is suitable for tropifexor drug interaction studies due to its good sample adaptability and high stability.

## Figures and Tables

**Table 1:** The mass spectral parameters, cone voltage (CV), collision energy (CE), and retention time (RTs) of tropifexor and ISTD.

Analyte	Parent ion	Daughter ion	CV (V)	CE (eV)	RT (min)
Tropifexor	604.08	228.03	20	30	1.53
ISTD	533.18	199.01	20	25	1.90

**Table 2:** The accuracy and precision of tropifexor in Beagle plasma (*n* = 6).

Added (ng/mL)	Intraday			Interday		
	Found (ng/mL)	RSD (%)	RE (%)	Found (ng/mL)	RSD (%)	RE (%)
1	0.99 ± 0.07	7.31	−1.00	0.95 ± 0.02	2.22	−4.89
2.5	2.50 ± 0.15	5.86	0.07	2.49 ± 0.06	2.43	−0.44
50	49.63 ± 2.14	4.31	−0.75	50.58 ± 0.57	1.14	1.16
150	149.74 ± 3.91	2.55	−0.17	150.66 ± 0.84	0.56	0.44

**Table 3:** Recovery and matrix effect of tropifexor from Beagle plasma (*n* = 6).

Added (ng/mL)	Recovery (%)	RSD (%)	Matrix effect (%)	RSD (%)
2.5	90.76 ± 2.34	2.58	99.88 ± 2.09	2.09
50	88.13 ± 1.80	2.05	101.44 ± 2.39	2.36
150	93.13 ± 1.42	1.53	101.93 ± 1.87	1.84

**Table 4:** Stability of tropifexor in beagle plasma subjected to various conditions (*n* = 6).

Added (ng/mL)	Room temperature, 4 h		Autosampler 24°C, 24 h		Three freeze–thaw		−80°C, 60 days	
	RSD (%)	RE (%)	RSD (%)	RE (%)	RSD (%)	RE (%)	RSD (%)	RE (%)
2.5	3.84	0.33	4.44	1.13	5.36	−5.80	4.49	−8.20
50	3.59	−0.97	3.45	0.64	4.87	1.78	2.45	−3.19
150	1.91	0.24	1.94	−0.03	2.57	−1.09	1.07	−2.66

**Table 5:** The major pharmacokinetic parameters ascribed to tropifexor in beagles (*n* = 6, Mean ± SD).

Parameters	Unit	Tropifexor
*C *_max_	ng/mL	76.87 ± 17.26
*T *_max_	h	3.33 ± 0.52
*t *_1/2_	h	9.27 ± 2.63
CLz/F	L/h/kg	0.53 ± 0.16
AUC_(0⟶t)_	ng·h/mL	664.52 ± 229.21
AUC_(0⟶∞)_	ng·h/mL	685.96 ± 232.57

## Data Availability

The original contributions presented in the study are included in the article; further inquiries can be directed to the corresponding author.
